# A Single Tube Overlap Extension PCR Method for Splicing of Multiple DNA Fragments

**Published:** 2020

**Authors:** Farzaneh Zarghampoor, Abbas Behzad-Behbahani, Negar Azarpira, Saeed Reza Khatami, Maryam Fanian, Mahdokht Hossein Aghdaie, Gholamreza Rafiei Dehbidi

**Affiliations:** 1. Department of Biology, Faculty of Science, Shahid Chamran University of Ahvaz, Ahvaz, Iran; 2. Diagnostic Laboratory Sciences and Technology Research Centre, Faculty of Paramedical, Shiraz Sciences University of Medical Sciences, Shiraz, Iran; 3. Transplant Research Centre, Shiraz University of Medical Science, Shiraz, Iran

**Keywords:** Beta-globins, Mutagenesis, Polymerase chain reaction, Site-directed, Untranslated regions

## Abstract

**Background:**

Despite the ease of conventional splicing by overlap-extension (SOEing) PCR technique in theory, when splicing more than two fragments, and especially if one of the complementary sequences is A-T rich, the attachment of the fragments would be challenging. A new rapid and highly efficient SOEing PCR assay was developed for simultaneous splicing of multiple DNA fragments and induction of site-directed mutagenesis in a single tube.

**Methods:**

The method was adapted for splicing human beta-globin UTRs to OCT4, SOX2, KLF4, C-MYC, LIN28A, and destabilized GFP for the construction of chimeric DNA fragments for *in vitro* transcription. In addition, the native Kozak sequence of beta-globin (K1) was replaced by the strongest Kozak sequence (K2) using site-directed mutagenesis to enhance the expression of target genes.

**Results:**

ChimericGFPd2/K1, GFPd2/K2, OCT4, and KLF4 were created by the optimized conventional SOEing PCR. The single tube method was able to create the chimeric SOX2, C-MYC, and LIN28A in high quality and quantity in comparison with the conventional SOEing PCR. Moreover, using single tube SOEing PCR, the reaction time and materials that are required in the conventional SOEing PCR were significantly reduced. Fluorescent microscopy and flow cytometry examinations indicated highly efficient translation of K2 sequence in comparison with the K1sequence.

**Conclusion:**

Single tube SOEing PCR is a valuable method to construct more multiple fragments with high yield. The method can successfully be applied for construction of various kinds of complex chimeric genes.

## Introduction

The Splicing by overlap-extension/Splicing by over-hang-extension PCR (SOEing PCR) is a type of PCR which is used to insert specific mutations at specific points in a sequence 1,2 or splice smaller DNA fragments to construct chimeric gene fragment with no dependence on the restriction site or ligase 3. Any overlap sequences or mismatches can be incorporated into the 5′end of primers, so that a DNA fragment with incorporate new sequences which did not exist in the first template is created. This idea was first introduced under the title of mispriming 4,5. Using simple mispriming can create site-directing mutagenesis only at the end of PCR products 6, whereas overlap-extension can generate mutations in the center of PCR-products 2.

To create chimeric DNA fragment by the conventional SOEing PCR, two separate PCR-generated products should have been a short overlap of complementary sequence (typically 30–60 *bp*). The overlap can be formed in the PCR products by addition of bases at the 5′ ends of primers. Subsequently, the fragments are combined in equal amounts of molecules of all fragments to create a longer fragment sequence. After splicing two fragments at the complementary sequence, the 3′ end of each fragment plays the role of primer and continues the extension. The resulting product is further amplified by PCR 6–8.

Despite the ease of the SOEing PCR technique in theory and its advantages compared with the other techniques such as restriction method, its complexity limits its application 7,8. When splicing more than two fragments, especially if the complementary sequence is AT-rich 9, splicing of fragments would be challenging. In addition, in the conventional SOEing PCR, the smear or multiple bands are often seen on the agarose gel electrophoresis and occasionally the main band is very weak. On the other hand, constructing a chimeric DNA fragment from multiple small fragments requires several PCR reactions, thus the conventional SOEing PCR is tedious, time-consuming, and not cost-effective. Furthermore, random error may be increased by the polymerase during several PCR reactions. Although some researchers have successfully assembled up to four DNA fragments simultaneously 10,11, multiple DNA splicing based on overlap extension PCR remains a challenge which needs to be improved.

In the present study, a single tube overlap extension PCR method was developed for the simultaneous fusion of 5′ and 3′ untranslated regions of human beta globin into classical reprogramming genes including SOX2, C-MYC, and LIN28A. Furthermore, to obtain the maximum production of chimeric DNA constructs including destabilized GFP, OCT4, and KLF4, the conventional SOEing PCR method was modified by improving parameters such as annealing temperature of the complementary sequences, reaction conditions, and elongation time. The chimeric DNA templates were then used for *in vitro* transcription and production of stable synthetic mRNA after transfection in the eukaryotic cells. In addition, the SOEing PCR technique was adapted to replace the native Kozak sequence (K1) of beta-globin by the strongest Kozak sequence (K2) to enhance the translation efficiency of the mRNA.

## Materials and Methods

In the present study, for splicing the UTRs of beta-globin gene to Reprogramming Factors (RFs) and GF-Pd2, the conventional and developed overlap extension PCR (single tube) were used.

### Primer designing for splicing of DNA fragments

The GeneRunner software (Version 6.5.51×64 Beta) was used to design the primers for the PCR amplification of different DNA fragments. The sequences of the nineteen set of primers used for this recombination are listed in table 1. The internal primers (the primer with a short overlap of complementary sequence) were designed between 20 and 33 *bp* for splicing the fragments together and generating site-directed mutation in the K1 sequence in order to create the K2 sequence. The primer specificity was confirmed by Primer-BLAST (www.ncbi.nlm.nih.gov/tools/primer-blast). The internal primers containing the K1 and K2 sequences were just designed for chimeric GFPd2 construct and the internal primers of RF contained the K2 sequence.

**Table 1.** Primers designed for amplifying fragments, the OE- PCR, and generation of the K2 90 sequence

**DNA fragment name**	**Primer name**	**Primer sequence**	**Product length (bp)**
**5′UTR**	5′UF 5′UR1	**5′tcaaggatcc**GATCAATAATACGACTCACTATAG3′ **5′ctcacc**ATGGTGTCTGTTTGAGGTTG 3′	176
	5′UF 5′UR2	**5′tcaaggatcc**GATCAATAATACGACTCACTATAG3′ **5′ tcccgccat**GGTGGCGGTTTGAG 3′	176
	5′UF 5′UR3	**5′tcaaggatcc**GATCAATAATACGACTCACTATAG3′ **5′tgttgtacat**GGTGGCGGTTTGAGG 3′	177
	5′UF 5′UR4	**5′tcaaggatcc**GATCAATAATACGACTCACTATAG3′ **5′gacagccat**GGTGGCGGTTTGAG 3′	176
	5′UF 5′UR5	**5′tcaaggatcc**GATCAATAATACGACTCACTATAG3′ **5′aggggcat**GGTGGCGGTTTGAGG 3′	175
	5′UF 5′UR6	**5′tcaaggatcc**GATCAATAATACGACTCACTATAG3′ **5′agcccat**GGTGGCGGTTTGAG 3′	174

**3′UTR**	3′UF1 3′UR	**5′caatgtgtag**GCTCGCTTTCTTGCTGTCC 3′ **5′ cacagaattc**GCTCTTCTTTTTGCAATG3′	166
	3′UF2 3′UR	**5′ cattcaaactga**GCTCGCTTTCTTGCTGTC3′ **5′ cacagaattc**GCTCTTCTTTTTGCAATG3′	166
	3′UF3 3′UR	**5′ cacatgtga**GCTCGCTTTCTTGCTGTC3′ **5′ cacagaattc**GCTCTTCTTTTTGCAATG3′	163
	3′UF4 3′UR	**5′gacatttttaa**GCTCGCTTTCTTGCTGTCC3′ **5′ cacagaattc**GCTCTTCTTTTTGCAATG3′	165
	3′UF5 3′UR	**5′ ttgtgcgtga**GCTCGCTTTCTTGCTGTC 3′ **5′ cacagaattc**GCTCTTCTTTTTGCAATG3′	164
	3′UF6 3′UR	**5′ acagaattga**GCTCGCTTTCTTGCTGTCC 3′ **5′ cacagaattc**GCTCTTCTTTTTGCAATG3′	164

**GFPd2**	GK1F GK1R	**5′ gacacc**ATGGTGAGCAAGGGCGAG 3′ **5′ gaaagcgagc**CTACACATTGATCCTAGCAGAAG 3′	853

**Modified Kozak**	5′UK2F 5′UK2R	**5′tcaaggatcc**GATCAATAATACGACTCACTATAG 3′ **5′cttgctcaccat**GGTGGCGGGTTGAGGTTG 3′	176

**GFPd2K2**	GK2F GK2R	**5′aacagacacc**ATGGTGAGCAAGGGCGAG 3′ **5′ gaaagcgagc**CTACACATTGATCCTAGCAGAAG	858

**OCT4**	OF OR	**5′ gccacc**ATGGCGGGACACCTG 3′ **5′ gaaagcgagc**TCAGTTTGAATGCATGG 3′	1099

**SOX2**	SF SR	**5′ aaccgccacc**ATGTACAACATGATG 3′ **5′ gaaagcgagc**TCACATGTGTGAGAGG 3′	974

**KLF4**	KF KR	**5′ ccgccacc**ATGGCTGTCAGTG 3′ **5′ gaaagcgagc**TTAAAAATGTCTCTTCATGTG 3′	1431

**C-MYC**	MF MR	**5′ cgccacc**ATGCCCCTCAACGTTAG 3′ **5′gaaagcgagc**TCACGCACAAGAGTTC 3′	1337

**LIN28A**	LF LR	**5′ cgccacc**ATGGGCTCCGTGTC 3′ **5′ aaagcgagc**TCAATTCTGTGCCTCC 3′	646

Key: Overlap of complementary sequence are shown in bold lowercase letters.

### Plasmids

PUC57 vector containing5′ and 3′ UTR of human beta-globin, T7 promoter for *in vitro* transcription and the native Kozak sequence (K1) of human beta-globin were synthesized (Biomatik Corporation, Canada). The plasmids of pMXs-hOCT4 12, pMXs-hSOX2 12, pMXs-hKLF4 12, pMXs-hcMYC 12, pMXs-hLIN28A 13, and pCAG-GFPd2 14 were purchased from Addgene, a US non-profit organization. The pcDNA 3.1+ plasmid (In-vitrogen) contains a CMV promoter serving as plasmid backbone for cloning of the chimeric DNA fragments.

### The simple PCR reaction conditions

The UTRs and gene fragments were separately amplified by the simple PCR method, using Q5® Hot Start High-Fidelity 2X Master Mix (New England Biolabs, U.K). The 5′UTR containing the K2 sequence was created by site-directed mutagenesis SOEing PCR. The PCR products were determined on agarose gel electrophoresis and then recovered from the gel by AccuPrep® Gel Purification Kit (Bioneer, South Korea) according to the manufacturer′s protocol. The simple PCR reactions were performed in a final volume of 20 *μL* of mixture containing 10 *μL* of master mix, 1 *μL* of the plasmid DNA, and 0.5 *μM* of each primer. The PCR reactions were performed in the following cycling conditions: 98*°C* for 2 *min*, 30 cycles at 98*°C* for 10 *s*, X*°C* for the 30 *s*, 72*°C* for 1 *min*, and a final elongation step at 72*°C* for 4 *min*. The annealing temperatures (X*°C*) for the PCR reactions were estimated using NEB Tm calculator (https://tmcalculator.neb.com/#!/main).

### Splicing by overlap extension (SOEing) polymerase chain reaction

In order to splice DNA fragments together, the annealing temperature of complementary sequence I and II (I: 5′UTR-gene; II: gene-3′UTR) was initially estimated. The chimeric DNA fragments including GFPd2, OCT4 and KLF4 were created by the conventional SOEing PCR and chimeric reprogramming factors including SOX2, C-MYC, and LIN28A created by the single tube SOEing PCR.

### The conventional SOEing PCR reaction conditions

To construct chimeric DNA fragments using the conventional SOEing PCR, the 3′UTR was initially ligated to RFs or GFPd2 (in the SOE 1 reaction) and then 5′UTR was ligated (in the SOE 2 reaction). To achieve this, the fragments were combined in equal amounts of molecules of two fragments without primers and the PCR mixture was subjected to PCR with the following cycling conditions, as illustrated in table 2. The annealing temperature for the ligation of the two fragments was determined with the complementary sequence of two fragments and using NEB Tm calculator. To carry out the steps 1 and 2 of the conventional SOE 1 reaction, the final volume of reaction mixture was 18.6 *μL* containing 2.5 *μL* 10X PFU buffers, 2.5–3 U PFU DNA polymerase, 0.1 *mM* dNTPs, and 2.5 *μL* of the DNA fragment mixture (372 *ng*/2 *μL* of GFPd2 + 67.64 *ng*/1.78 *μL* of 3′UTR). In step 3, 0.5 *μM* of primers was added into the mixture and the steps 4 and 5 of PCR were performed. To confirm the ligation of the two DNA fragments, PCR products were run on agarose gel electrophoresis and were then purified from agarose gel. Using the SOE 2 reaction, 5′UTR fragment was ligated to the gene-3′UTR fragment with the same procedures. The 5′UTR/K2-GFPd2-3′UTR was also constructed using the conventional SOEing PCR method.

**Table 2.** The OE 1 reaction for construction GFPd2-3′UTR fragment

**Step**	**Cycle**	**Denaturation**	**Annealing**	**Extension**
**1**	1	95*°C* for 3 *min*	--	--
**2**	12	95*°C* for 30 *s*	55*°C* for 45 *s*	72*°C* for 2 *min*
**3**	1	50*°C* for 6 *min*	--	--
**4**	30	95*°C* for 30 *s*	55*°C* for 45 *s*	72*°C* for 2 *min*
**5**	1	--	--	72*°C* 5 *min*

### The single tube SOEing PCR reaction conditions

In a single tube SOEing PCR, the fragments were combined in equal amounts of molecules of three fragments without primers and the mixture was subjected to PCR with the following cycling conditions illustrated in table 3. The steps 1 and 2 of the single tube SOEing PCR reactions were carried out in a final volume of 28 *μL* of the mixture containing 5 *μL* 10X PFU buffers, 6 U PFU DNA polymerase, 0.2 *mM* dNTPs and 4 *μL* of the DNA fragment mixture. In step 3, 0.3 *μM* of external primers (5′UF and 3′UR) were added into the mixture and the steps 4 and 5 of PCR were performed.

**Table 3.** The single tube OE- PCR reaction for construction of 5′UTR+ K2-SOX2/C-MYC/LIN28A-3′UTR

**Step**	**Cycle**	**Denaturation**	**Annealing**	**Extension**
**1**	1	95*°C* for 4 *min*	--	--
**2**	13	95*°C* for 30 *s*	X*°C* for 55 *s*	72*°C* for 3 *min*
**3**	1	50*°C* for 6 *min*	--	--
**4**	32	95*°C* for 30 *s*	55*°C* for 50 *s*	72*°C* for 3 *min*
**5**	1	--	--	72*°C* 5 *min*

### Cloning of the chimeric fragments

The full-length chimeric DNA constructs were excised and purified from the agarose gel and then weredouble digested using EcoRI (New England Biolabs, U.K) and BamHI (New England Biolabs, U.K) restriction enzymes. The constructs were then inserted into pcDNA™3.1 (+) mammalian expression vector (Invitrogen) using calcium chloride method and the recombinant plasmids were transformed into the *Escherichia coli* (*E. coli*) strain DH5α. To screen the plasmids containing the desired inserted DNA, colony PCR was performed. For further confirmation, DNA sequencing was performed on all plasmids containing chimeric genes.

### Cell culture and plasmid DNA transfection

The HEK293T cell line was cultured in DMEM supplemented with 10% FBS (Gibco, USA) and incubated at 37*°C* in 5% CO_2_. For transfection, the cells were in the 3rd passage. The cells were seeded (5×104 HEK293T cells/well) in 24-well plates a day prior to the experiment. The chimeric GFPd2/K1 or K2 plasmid transfections were performed with Lipofectamine 3000 (Invitrogen). Hence, the culture media was changed to Opti-MEM I Reduced Serum Media (Thermo Fisher Scientific) 2 *hr* before transfection. The transfection was performed according to the manufacturer′s protocol and then the mixture was added to the culture media and incubated at 37*°C* in 5% CO_2_. Four hours after transfection, the culture media was changed to DMEM supplemented with 10% FBS. A day post-transfection, the cells were monitored under a fluorescence microscope.

### Flow cytometry assay

The HEK293T cell line was trypsinized 24 *hr* post-transfection and washed twice with PBS. Then, the cells were suspended in PBS and analyzed by flow cytometry method (BD FACS Calibur).

## Results

### Construction of the chimeric DNA fragments

**Figure 1.**
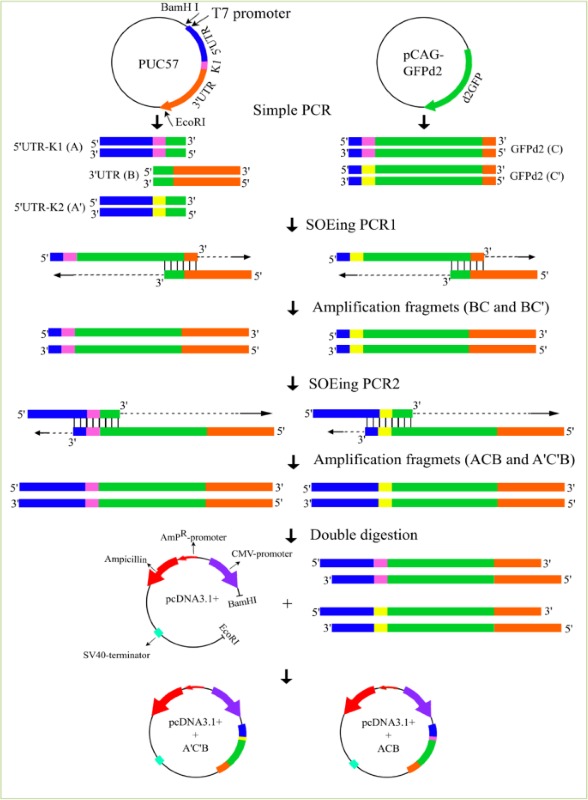
Construction of chimeric GFPd2 fragments using the conventional OE- PCR. In the first stage, the 5′UTR-K1 (A), 5′UTR-K2 (A′), 3′UTR (B), GFPd2 with K1 sequence (C), and GFPd2 with K2 sequence (C′) fragments were amplified using the simple PCR. The fragments had a complementary overlapping end to ensure splicing fragments together. The complementary sequence II was AT-rich; thus, the OE 1 reaction was initially performed for ligation of 3′UTR to GFPd2. In the OE 1 reaction, fragment (B) spliced to fragments (C and C′) and created the new chimeric fragments (CB and C′B). The 3′end of each fragment plays the role of primer and continues extension. The resulting products were amplified further by PCR. Following, the fragments (A and A′) spliced to fragments (CB and C′B) using the OE 2 reaction and created the new chimeric fragments (ACB and A′C′B) as well. Finally, the fragments (ACB and A′C′B) were amplified with outside primers. The final chimeric constructs double digested using EcoRI and BamHI restriction enzymes; then, the constructs were inserted into pcDNA™3.1 (+) Vector by Calcium chloride method.

**Figure 2.**
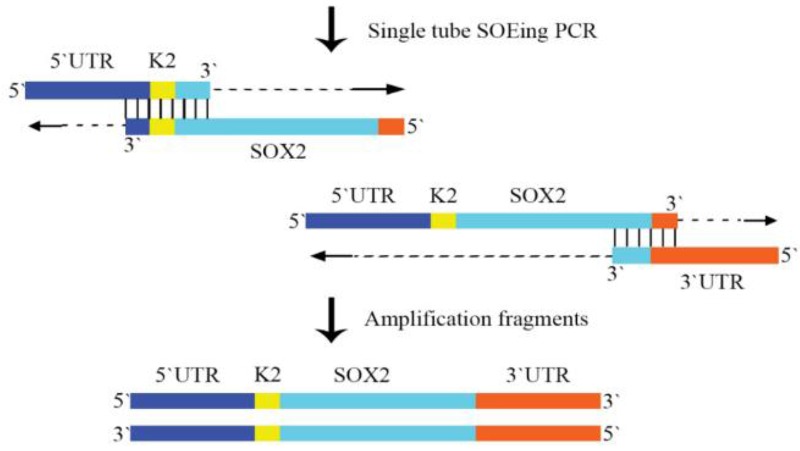
The principle of construction of the chimeric SOX2 fragment using the single tube OE- PCR. The complementary sequence I and II of SOX2 had the same annealing temperature; therefore, the chimeric DNA fragment was constructed using the single tube OE- PCR method. The 3′end of each fragment plays the role of primer and continues extension. The resulting products were amplified with the outside primer.

**Figure 3.**
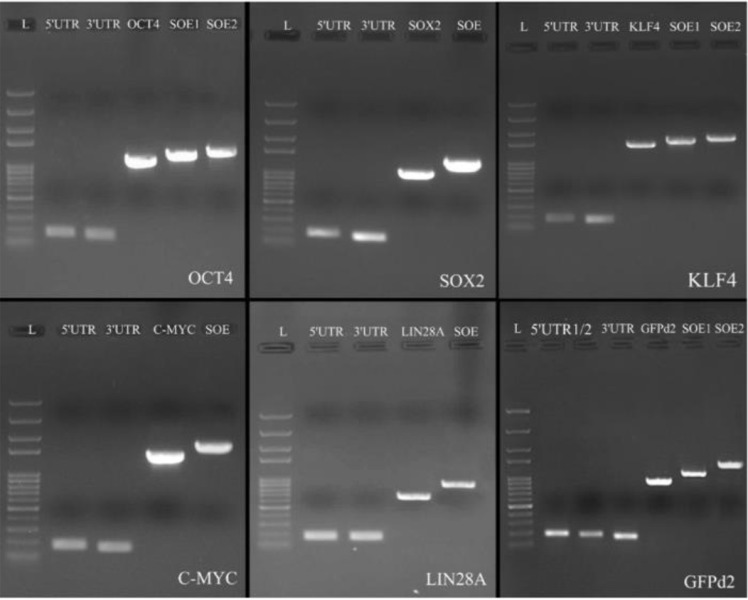
Construction of chimeric reprogramming factors and GFP-d2. OCT4: 100 *bp*-5 *Kb* DNA ladder (L), 5′UTR (176 *bp*), 3′UTR (166 *bp*), OCT4 fragment (1099 *bp*), OE 1 (1260 *bp*), OE 2 (1404 *bp*). SOX2: 100 *bp*-5 *Kb* DNA ladder (L), 5′UTR (177 *bp*), 3′UTR (163 *bp*), SOX2 fragment (974 *bp*), single tube OE- PCR (1275 *bp*). KLF4: 100 *bp*-5 *Kb* DNA ladder (L), 5′UTR (176 *bp*), 3′UTR (165 *bp*), KLF4 fragment (1431 *bp*), OE 1 (1590 *bp*), OE 2 (1734 *bp*). C-MYC: 100 *bp*-5 *Kb* DNA ladder (L), 5′UTR (175 *bp*), 3′UTR (164 *bp*), C-MYC fragment (1337 *bp*), single tube OE- PCR (1641 *bp*). LIN28A: 100 *bp*-5 *Kb* DNA ladder (L), 5′UTR (174 *bp*), 3′UTR (164 *bp*), LIN28A fragment (646 *bp*), single tube OE- PCR (951 *bp*). GFPd2: 100 *bp*-5 *Kb* DNA ladder (L), 5′UTR-k1 (176 *bp*), 5′UTR-k2 (176 *bp*), 3′UTR (166 *bp*), GFPd2 fragment (853 *bp*), OE 1 (1011 *bp*), OE 2(1147 *bp*).

In order to splice DNA fragments, the GC content and temperature of the complementary sequences were initially evaluated. The annealing temperature of complementary sequence I and II of SOX2, C-MYC, and LIN28A was the same while the annealing temperature of complementary sequence I and II of GFPd2, OCT4 and KLF4 was different. Thus, the chimeric DNA fragments including GFPd2, OCT4 and KLF4 were created by the conventional SOEing PCR (Figure 1) and chimeric reprogramming factors including SOX2, C-MYC, and LIN28A were created by the single tube SOEing PCR (Figure 2). In addition, the K1 sequence (ACAGACACCATG) was successfully replaced by K2 sequence (GCCGCCACCATG) using SOEing PCR to enhance translation efficiency. Surprisingly, the concentration of purified chimeric SOX2, C-MYC, and LIN28A was higher than GFPd2, OCT4, and KLF4 (for example, SOX2: 358 *ng/μL vs.* GFPd2: 52 *ng/μL*). Figure 3 shows the PCR products of simple PCRs and the SOEing PCRs of reprogramming factors and GFP-d2 on agarose gel electrophoresis.

### Plasmid DNA transfection and flow cytometry assay

**Figure 4.**
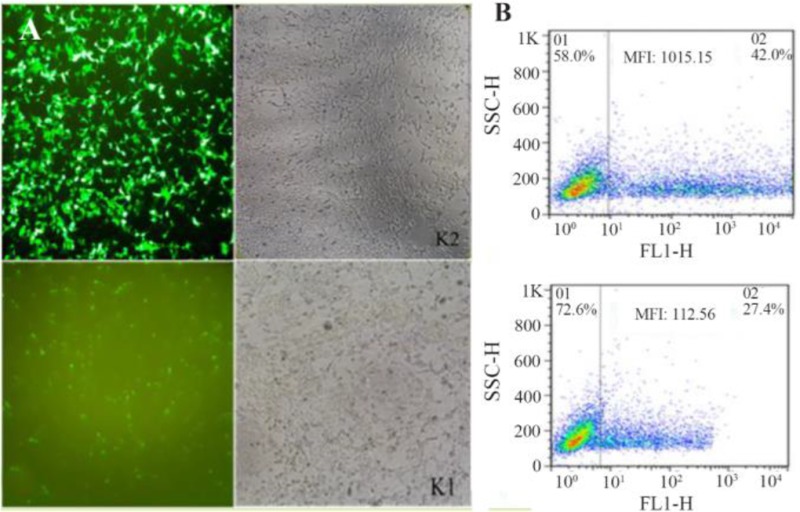
Transfection using Lipofectamine 3000. (A): The transfection of chimeric GFPd2 with the K2 and K1 sequence in HEK293T cell line after 24 *hr* illustrated by fluorescent microscope. (B): The mean fluorescent intensity (MFI) of GFP expression from chimeric GFPd2-K2 or K1 plasmid 24 *hr* after transfection.

To evaluate the performance of Kozak sequences in the efficiency of translation, both chimeric GFPd2 constructs were transfected into HEK293T cells using Lipofectamine 3000. The results obtained from fluorescent microscope 24 *hr* post-transfection showed that GFP production from the K2 plasmid was significantly more than the K1 plasmid. On the other hand, the transfected cells with the K2 plasmid initiated GFP expression earlier than the K1 plasmid. The GFP expression intensity of both plasmids was evaluated by flow cytometry assay as well. Overall, the mean fluorescence intensity of the K2 plasmid was higher than the K1 plasmid. Hence, other chimeric genes were constructed with the K2 sequence (Figure 4).

## Discussion

There are three procedures to construct a chimeric DNA fragment, including gene synthesis, the restriction method and the SOEing PCR strategy. Compared with the SOEing PCR approach, the restriction method needs the integration of mutations to construct unique restriction sites. Thus, the restriction method cannot be made at any position within the nucleotide sequence. On the other hand, construction of the unique restriction site requires introduction of an unwanted sequence into the junction sites. Nevertheless, the SOEing PCR successfully achieved gene ligation, using the polymerase chain reaction at any chosen location devoid of unwanted sequence 15. Another method to construct a chimeric DNA fragment is gene synthesis but for construction, the large chimeric DNA fragment is not cost-effective.

The conventional SOEing PCR method included several PCR reactions and requires laborious steps to purify the chimeric intermediate fragments to construct the final recombinant fragments. Thus, this method is tedious, time-consuming, expensive, and may increase mutations in the sequence of DNA fragments. Despite success to assemble more than three DNA fragments simultaneously 16,17, multiple DNA splicing in a single tube remains a challenge for researchers. A single tube SOEing PCR method was successfully developed in producing the desired chimeric fragments and induction of site-directed mutation. This OE-PCR method is distinct from previous gene assembly approaches 18,19 in that the parameters such as annealing temperature of the complementary sequences, reaction conditions, and elongation time were improved.

The induced Pluripotent Stem Cells (iPSCs) hold promise in the field of regenerative medicine, tissue bioengineering, disease modeling, autologous cell ther apy, and basic research. There are several procedures can be successfully generated iPSCs 19. Of those, IVT mRNA-reprogramming has several advantages compared with the other procedures 20–22. However, IVT mRNA has several limitations to be overcome. The main limitation of IVT mRNA is short half-life mRNA and mRNA-mediated translation. One approach for increasing IVT mRNA stability and protein translation is used 5′ and 3′ UTR of genes that have long half-life to construct the chimeric reprogramming factors. There-fore, in the present study spliced the 5′ and 3′UTRs of human beta-globin to GFPd2 and RFs to construct *in vitro* transcription DNA template. Additionally, to further enhance protein production, the Kozak sequence (K1) of the 5′UTR beta-globin gene replaced by the strongest Kozak sequence (K2) by the site-directed mutagenesis OE- PCR as well.

Concentration, ratio, and purity of initial fragments are the key factors influencing the efficacy of the SOEing PCR reaction. The fragment concentration ratio is the inverse of the fragment size ratio. Consequently, in the present study, the conventional SOEing PCR operational conditions such as regulation of PCR conditions (including Tm, time of the extension, ratio, and concentration of the initial DNA template) were optimized and a single tube SOEing PCR was introduced.

In the conventional SOEing PCR (For example, to construct chimeric 5′UTR-GFPd2-3′UTR), three fragments were amplified which had a short overlap of the complementary sequence. Subsequently, SOE 1 and 2 reaction was performed for generation of GFPd2-3′UTR and 5′UTR-GFPd2-3′UTR, respectively. It was found that for ligation of different fragments, Tm and GC% of the complementary sequence should first be calculated. The annealing temperature of the complementary sequence of the two fragments was estimated, using NEB Tm calculator or according to the formula Td=4(C+G)+2(A+T) in Celsius degrees. In the SOE reaction, the fragments that had low Tm or GC% complementary sequence were preferentially spliced. It is critical to use PFU DNA polymerase for the SOEing PCR because Taq DNA polymerase may create mutations in the sequence, and especially in the case of *in vitro* transcription, the DNA template should be error-free. Thus, Q5 Hot Start High-Fidelity 2X Master Mix was used for amplification. However, it was found that it would be better to use PFU DNA polymerase in the ligation steps and increase the extension time up to 2–3 *min*. Interestingly, it was found that if the annealing temperature of complementary sequence I and II is the same, SOEing PCR can be used in the single tube reaction. Therefore, ligation of UTRs to SOX2, C-MYC, and Lin28A was performed in the single tube SOEing PCR reaction.

In cases of GFPd2, OCT4 and KLF4, the annealing temperature of complementary sequence I and II was different. Hence, these chimeric fragments were constructed using the optimized conventional SOEing PCR. In each case, the chimeric DNA fragments were successfully generated. However, the concentration of purified chimeric DNA fragments from the single tube SOEing PCR was significantly higher than the conventional SOEing PCR procedures. The method provided a maximum efficiency yield of chimeric DNA fragments in minimum time, reduced the steps in the procedure, and was cost effective compared with the conventional method. Thus, in designing the internal primers (primer with overlap sequence), the annealing temperature of complementary sequences should be the same or similar so that the SOEing PCR can be performed in single tube procedures. However, in order to construct complex chimeric DNA fragments with different annealing temperatures of complementary sequence, it is suggested to perform touch-up SOEing PCR method.

## Conclusion

To sum up, the current study described a very reliable and efficient SOEing PCR procedure that allows a quick construction of chimeric DNA fragments. The procedure allows to combine more than two DNA fragments simultaneously in a single reaction tube. The method prevents the risk of undesirable mutation which usually occurs when DNA polymerase is used. The efficiency and simplicity of this procedure makes it a valuable approach for generating chimeric fragments in genetic engineering studies.
